# Genome-Wide Association Study Identifies Candidate Genes for Stripe Pattern Feather Color of Rhode Island Red Chicks

**DOI:** 10.3390/genes13091511

**Published:** 2022-08-24

**Authors:** Qingmiao Shen, Jieke Zhou, Junying Li, Xiaoyu Zhao, Lijie Zheng, Haigang Bao, Changxin Wu

**Affiliations:** 1 National Engineering Laboratory for Animal Breeding, College of Animal Science and Technology, China Agricultural University, Beijing 100193, China; 2 Dawu Breeding Company, Baoding 072550, China

**Keywords:** Rhode Island Red chicks, feather color, stripe pattern, GWAS, *KITLG*, *TMTC3*

## Abstract

Feather colors of chickens are not only characteristics of breeds but also as phenotypic markers in chicken breeding. Pure-bred Rhode Island Red (RIR) chicks have a stripe pattern and a non-stripe pattern on the back. The stripe pattern of RIR is generally shown as four longitudinal black stripes on the back and is more likely to appear in females. In this study, we performed a genome-wide association study (GWAS) to identify candidate genes controlling the stripe pattern of RIR chicks, and then, based on physical location and biological functions, quantitative RT-PCR analysis was used to validate the differential expression of candidate genes between stripe pattern and non-stripe pattern back skin tissue. The GWAS showed that a major signal contains 768 significant single nucleotide polymorphisms (SNPs) and 87 significant small insertions-deletions (INDELs) spanning 41.78 to 43.05 Mb (~1.27 Mb) on GGA1, corresponding to 16 genes associated with stripe pattern phenotype. Among these 16 genes, *KITLG* and *TMTC3* could be considered candidate genes as they showed different expressions between back skin tissues of stripe pattern and non-stripe pattern chicks in value (*p* = 0.062) and the significant level (*p* < 0.05), respectively. This study provided novel insight into the mechanisms underlying feather pigmentation and stripe formation in RIR chicks.

## 1. Introduction

Feather colors are not only characteristics of chicken breeds but also as phenotypic markers in chicken breeding. They can be categorized as patterned (dorsal and ventral pigmentation, spots, stripes, patches, etc.) and non-patterned (solid colored from heavily pigmented to white) at the whole-body level [1,2]. Over a long period of domestication, variations of feather color arose and was selectively bred, which led to a bewildering array of colors and patterns in chickens [3,4,5]. Melanin, including eumelanin (brown to black) and pheomelanin (yellow to red), was produced by melanocytes in hair follicles [3,6]. Feather colors are directly determined by the distribution of melanin type and density which depend on a cascade of molecular signal pathways during the complex processes of the regulation of melanocytes and melanin production [1,6,7]. In addition, the structural color, namely the interaction between the feather microstructure and light, also plays an important role in the final formation of the feather color [8,9,10].

Genes that control feather colors and their associated inheritance patterns in chickens have been extensively studied. Kerje et al. reported that the *MC1R* gene should be equal to the extended black (E) locus, and its mutations are related to chicken feather colors [11]. Mutations of *PMEL17* and *TYR* were responsible for dominant white and recessive white phenotypes in chicken, respectively [12,13]. Gunnarsson et al. found that two independent missense mutations (Tyr277Cys and Leu347Met) in *SLC45A2* were associated with the sex-linked silver locus (S) in chicken [14]. Thalmann et al. suggested that mutations in the regulatory region of *CDKN2A* cause sex-linked barring in chicken, and two variants in the CDS region of the same gene make the barring pattern more distinct independently [15]. Gunnarsson et al. demonstrated that an 8.3 kb deletion upstream of *SOX10* causes dark brown feather color in chickens [16].

**Figure 1:**
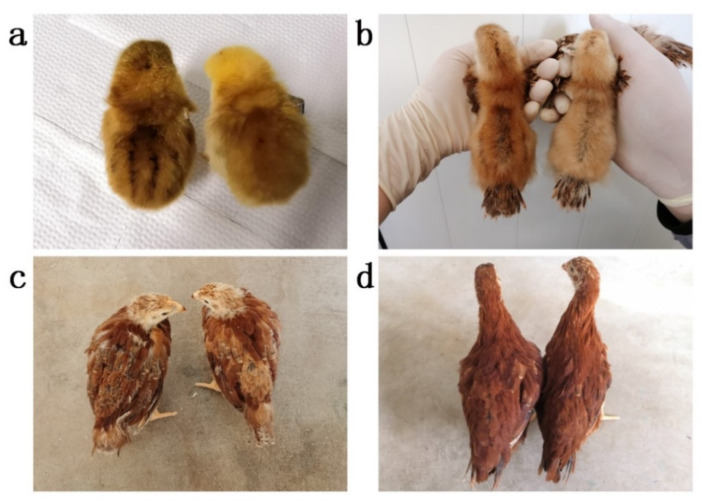
Stripe pattern and non-stripe pattern female RIR chickens of different ages. (**a**) 1-day-old; (**b**) 13-day-old; (**c**) 28-day-old; (**d**) 46-day-old. In each picture, the stripe pattern and non-stripe pattern are left and right, respectively. As the chick grows, the downy feathers are gradually replaced with youth feathers and the stripe pattern disappears.

Stripe patterns are the most prominent pigment patterns and often show on the back skin at the embryonic and juvenile stages of Galliformes birds [2]. It was reported that melanoblasts committed to producing eumelanin and formed longitudinal black stripes on the back of wild-type quail embryos before the apparent expression of melanogenic genes in melanocytes [17]. In the back derma of Galliformes embryos, expression patterns of *ASIP* were related to longitudinal stripe patterns (alternating yellow and black dorsal stripes) and regulated the width of yellow stripes [18,19]. Rhode Island Red (RIR) chicken is one of the most common breeds in the world and is often used as a cross parent for many commercial layers [4]. Pure-bred RIR chicks show stripe patterns and non-stripe patterns on the back (Figure 1a,b). The stripe pattern is generally shown as four longitudinal black stripes covering the back and is more likely to appear in female chicks younger than 2 weeks old. As the chick grows, the downy feathers are gradually replaced with youth feathers and the stripes disappear (Figure 1c,d). To date, the molecular mechanisms underlying the stripe pattern in RIR chicks remain unknown. We observed that in Dawu Breeding Company stripe pattern in females accounted for about 85–90% of the total female chicks, while in males, about 5% of the total male chicks. In this study, we used a pure-bred RIR chicken population to identify the candidate genes controlling stripe patterns while providing some clues for revealing the molecular mechanisms of the formation of black stripe patterns in chicks.

## 2. Materials and Methods

### 2.1. Animals and Sample Collection

All birds used in this study were from a pure-bred RIR population raised in Dawu Breeding Company (Baoding, China). Based on pedigree records, 14 roosters and 132 hens with no relationship between any two birds within two generations were selected from the pure-bred RIR population at the age of 30 weeks to breed their chicks, each rooster mating with 8–10 hens. Feather colors of chicks were distinguished within one week after hatching. Once hatched, a total of 74 female chicks, including 37 with the stripe pattern and 37 with the non-stripe pattern, were selected for a genome-wide association study (GWAS) according to the principle of full-sib or half-sib pairing. A blood sample of each female chick for GWAS was collected from the wing vein using 1 mL injectors at 8 weeks of age.

### 2.2. Whole-Genome Sequencing and Variant Calling

Genomic DNA was isolated from the 74 blood samples using the TIANamp Genomic DNA Kit (Cat. #DP304-03, TIANGEN Biotech (Beijing) Co., Ltd., Beijing, China) according to the manufacturer’s instructions. After being checked and qualified, DNA samples were delivered to a commercial company for next-generation sequencing. The whole-genome resequencing data were generated on Illumina NovaSeq 6000 platform with 150 bp paired-end reads. The average depth of resequencing for each sample was greater than 10 X. After removing reads with low-quality bases containing adapters or poly-Ns from raw data; the clean data were aligned against the reference genome sequence (GRCg6a) supported by Ensembl using the Bowtie 2 (version 2.4.5) with parameters “-p 8 -reorder -X 500”, and then sorted by SAMtools (version 1.11) [20,21]. Genome-wide single nucleotide polymorphisms (SNPs) and small insertions-deletions (INDELs) were detected by SAMtools (version 1.11) “mpileup” module and BCFtools (version 1.11) “call” option [21].

### 2.3. Genome-Wide Association Studies

VCFtools (version 0.1.16) was performed to filtering variants (SNPs and INDELs) with the following criteria: only bi-allelic sites, quality value per site > 30, mean depth value per site > 5, minor allele frequency > 0.05, missing rate per site < 0.1, distance between adjacent sites > 500 bp [22]. PLINK (version 1.90) was performed to filtering individuals genotype rate > 0.9 and Hardy–Weinberg equilibrium at *p* > 0.000001 [23]. After filtering, 74 chickens with 1,080,642 SNPs and 106,058 INDELs were retained. GWAS was performed by the “assoc” model of PLINK (version 1.90) software with 37 chicks of stripe pattern (case group) and 37 chicks of the non-stripe pattern (control group) [23]. The significance threshold for GWAS was set at 0.05 after correction for multiple tests by the FDR_BH method [24]. The Manhattan plot was drawn using the R package of qqman [25].

### 2.4. Variation Annotation and Candidate Gene Identification

The significant SNPs and INDELs were annotated to the gene region or within 5 kb upstream or downstream of the gene by snpEff software (version 4.5) based on the GRCg6a assembly supported by Ensembl [26]. Candidate genes for stripe patterns were identified based on the physical locations of the significant variations and biological functions of corresponding genes.

### 2.5. Quantitative Real-Time PCR

Twelve female chicks of one-day-old (6 birds per phenotype) were selected at random and a piece of back skin tissue of each chick was collected and immediately placed in liquid nitrogen. Total RNA was isolated using the Trizol protocol [27]. The quality and concentration were determined by NanoDrop 2000 Spectrophotometer (Thermo Fisher Scientific Inc., Waltham, MA, USA) and agarose gel (1.0%) electrophoresis. About 1 µg RNA of each sample was used for cDNA synthesis using a reverse transcription kit (Cat. #KR116-02, TIANGEN Biotech (Beijing) Co., Ltd., Beijing, China). In the differential expression analysis of two candidate genes of *TMTC3* and *KITLG* between chicks of the stripe pattern and the non-stripe pattern by quantitative Real-Time PCR (qRT-PCR) analyses, *GAPDH* was set as a reference control [28]. Primer sequences were designed using Primer 5.0 (PREMIER Biosoft, San Francisco, CA, USA) and are shown in Table 1. qRT-PCR was performed on Bio-Rad CFX96TM Real-Time System (Bio-Rad Laboratories, Inc., Hercules, CA, USA) with a 20 µL reaction system. Each sample had three biological replicates. The 20 µL of qRT-PCR reaction mixture contained 10 µL of 2 × SuperReal PreMix Plus (SYBR Green) (Cat. #FP205-02, TIANGEN Biotech (Beijing) Co., Ltd., Beijing, China), 0.6 µL of the forward primer (10 pmoL/μL), 0.6 μL of the reverse primer (10 pmoL/µL), 1 µL of cDNA template and 7.8 µL of RNase free water. The thermal cycling process was as follows: 95 °C for 15 min, 40 cycles of amplification (95 °C for 10 s, Tm for 30 s, and 72 °C for 30 s). Relative expression quantification of each gene was calculated by the 2^−ΔΔCt^ method [29]. The variance analysis was performed with SPSS software 21.0 (IBM Corp, Armonk, NY, USA), and the statistical significance level was set at *p* < 0.05.

## 3. Results

### 3.1. Overview of the Whole-Genome Sequencing Data

A summary of the whole-genome sequencing data is shown in Table S1. A total of 1821 G raw bases were obtained. After filtering, 1816 G clean bases were aligned with the genome reference of chicken (GRCg6a), and the Q20 value of each sample was above 95.2%. The alignment rate of the clean data of each sample was above 91.8%. These results showed that the sequencing data were of good quality and could be used for subsequent analyses.

### 3.2. Genome-Wide Association Studies

A total of 14,696,437 variants, including 11,517,331 SNPs and 3,179,106 INDELs, were identified in the present study (Table S2). After filtration, only 1,186,700 bi-allelic variants throughout the whole genome were used for the GWAS.

**Figure 2:**
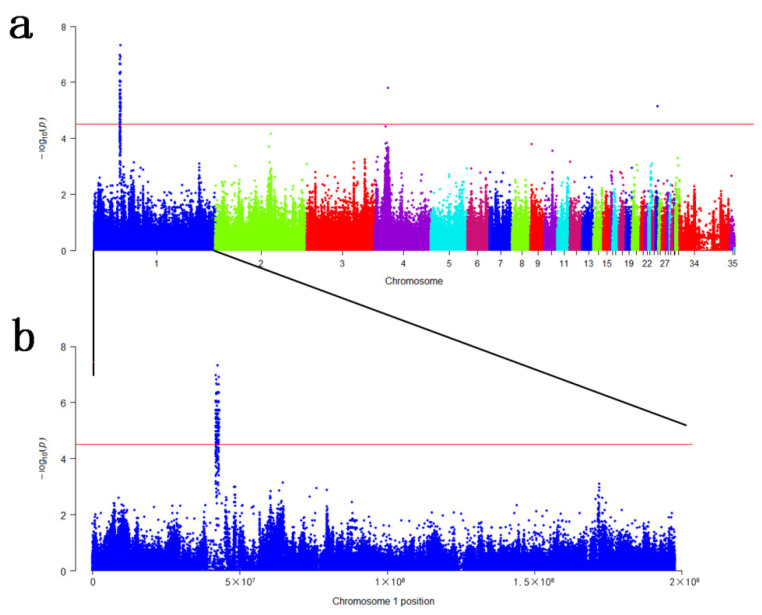
Manhattan plots of GWAS for RIR stripe pattern. (**a**) Manhattan plot of all association bi-allelic variants (SNPs and INDELs) with the RIR stripe pattern; (**b**) Manhattan plot of GGA1 association bi-allelic variants (SNPs and INDELs) with the RIR stripe pattern. Manhattan plots indicate -log10(*p*) for variants (y-axis) against their positions on each chromosome (x-axis). Chromosomes 34 and 35 indicate Chromosome Z and W, respectively. The solid red line represents the genome-wide significant threshold (*p* = 3.07 × 10^−5^).

GWAS revealed that 857 bi-allelic variants were associated with the RIR stripe pattern significantly (*p* < 3.07 × 10^−5^). The Manhattan plot is shown in Figure 2. A major association signal contains 768 SNPs and 87 INDELs were observed spanning a region about 1.27 Mb from the position of 41.78 Mb to 43.05 Mb on GGA1, corresponding to 16 genes, namely *TSPAN19*, *ENSGALG00000044478*, *ALX1*, *ENSGALG00000047575*, *RASSF9*, *NTS*, *MGAT4C*, *ENSGALG00000045907*, *ENSGALG00000053372*, *C12orf50*, *C12orf29*, *ENSGALG00000049176*, *ENSGALG00000051263*, *ENSGALG00000011177*, *TMTC3*, *KITLG* (Table 2). Besides, the other two significant SNPs were located on GGA 4 and GGA 25, respectively, corresponding to *ENSGALG00000048717*, *GASK1B,* and *KCNN3*. The descriptive summary of associated variants is shown in Table 2, and detailed information is provided in Table S3.

### 3.3. Quantitative Real-Time PCR

**Figure 3:**
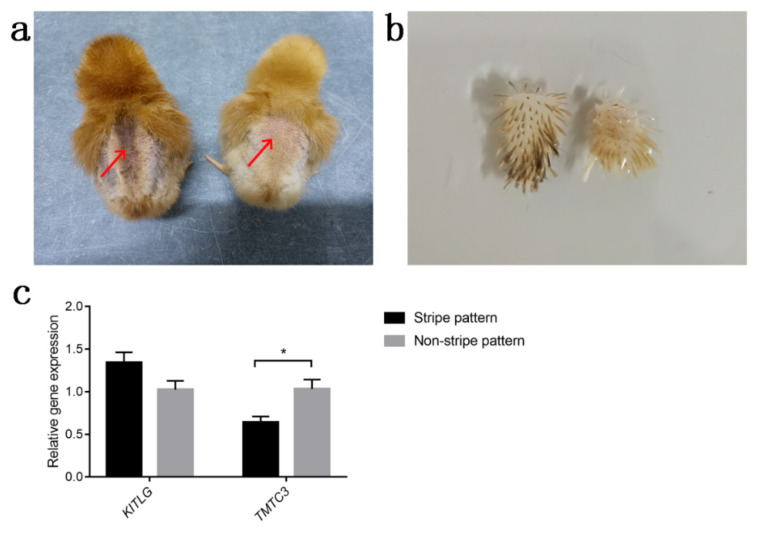
Relative expression of candidate genes in dorsal skin tissue of 1-day-old stripe and non-stripe pattern female RIR chicks. (**a**) The dorsal skin tissue collection location (red arrows) of the 1-day-old stripe pattern (left) and non-stripe pattern (right) RIR chicks; (**b**) Skin tissue collected from the stripe pattern (left) and non-stripe pattern (right); (**c**) Relative expression of *KITLG* and *TMTC3*. * represents *p* < 0.05.

Based on the results of GWAS and the biological functions of candidate genes, *KITLG* and *TMTC3* were considered as candidate genes for stripe patterns in the RIR chick dorsum. We used qRT-PCR to measure the relative expression of *KITLG* and *TMTC3* in dorsal skin tissue. The results indicated that the expression level of *TMTC3* was significantly higher in chicks of the stripe pattern than those of the non-stripe pattern (*p* = 0.021), and *KITLG* expression showed a downward trend from stripe pattern to non-stripe pattern chicks (*p* = 0.062) as shown in Figure 3.

## 4. Discussions

Although studies in feather color patterns of chickens have revealed some genetic and molecular mechanisms, the genes involved in a dorsal stripe pattern in RIR chicks is still unclear [2,4,30]. In this study, we perform a standard case/control association analysis using 74 RIR female chicks with a stripe or non-stripe pattern to identify candidate genes associated with dorsal stripes. Since the genetic background of the population is generally required to be consistent or similar between the case and control populations to avoid population stratification and reduce false positives [31], the sib-pair design was used in the present study to reduce the difference in genetic background between the case and control populations.

The Manhattan plots of GWAS are shown in Figure 2. As we can see from Figure 2 and Table 2, association signals are mainly in the genomic region ranging from 41.78 to 43.05 Mb (~1.27 Mb) on GGA 1. Although there is one significant SNP associated with stripe pattern on GGA 4 and GGA 25, respectively, there are no other significant signals nearby. Therefore, we mainly focused on the association region on GGA 1, which corresponded to 16 genes, including nine known genes and seven anonymous genes (Table 2).

The biological functions of the nine known genes are listed in Table 3. *KITLG* is the ligand of receptor tyrosine kinases (*KIT*), also known as stem cell factor (*SCF*). It was reported that *KIT/KITLG* signaling plays an essential role in melanoblasts/melanocytes proliferation, differentiation, migration, colonization, melanin production, gametogenesis, and hematopoiesis [32,33,34,35,36,37]. Some pigmentation disorders in humans are thought to be caused by *KITLG* mutations, such as Waardenburg syndrome type 2, as well as familial progressive hyper- and hypopigmentation [38,39,40]. Several variants in the upstream sequence of *KITLG* have been reported to be related to hair and coat color in different animals [41,42,43]. An SNP located in the upstream of *KITLG* was significantly associated with blond hair color in Iceland and Dutch [41]. In mice, an upstream inversion of the *KITLG* gene reduces hair pigmentation [42]. In the domestic dog, the copy number variant in the upstream of *KITLG* is responsible for coat pigment [43]. Furthermore, the genomic analysis suggested that *KITLG* be associated with the roan pattern in Pakistani goats [44]. In the present study, more than 10 SNPs in or nearby *KITLG* are significantly associated with the stripe pattern in the chick dorsum (Table S3). Therefore, we suggest that *KITLG* be one of the important candidate genes for the RIR stripe pattern.

*TMTC3* (transmembrane and tetratricopeptide repeat containing 3) was involved in some neuronal cell migration diseases in humans, such as cobblestone lissencephaly [45]. TMTC3 protein bonded to E-cadherin and enhanced cellular adherence, which played roles in cell migration and embryonic development [46]. Melanocytes and melanoblasts are derived from the neural crest; their adhesion to surrounding cells affects their migration to destinations of the dermis layer, epidermis, and hair follicles [56]. Melanoblasts produce eumelanin before melanogenic gene expression in melanocytes at early embryonic development [17,56]. E-cadherin, mainly expressed in the epidermis, plays an important role in the colonization of epidermal melanoblasts/melanocytes [56]. Therefore, we hypothesized that *TMTC3* affects the migration of melanoblasts resulting in pigmentation changes by its regulation of E-cadherin adhesion and suggested that *TMTC3* be another important candidate gene for chick stripe pattern in this study.

Except for *KITLG* and *TMTC3*, the rest of the seven known genes do not appear to be functionally related to chick feather colors (Table 3) [47,48,49,50,51,52,53,54,55]. *TSPAN19* was associated with plasma inhibin B levels [47]. *ALX1* affected craniofacial development and was also closely related to beak shape in Darwin’s finches [48]. *RASSF9* plays a role in regulating tumor proliferation and maintaining epidermal homeostasis [49,50,51]. *NTS* is a neuropeptide that is involved in the regulation of the central nervous system and digestive system and promotes tumor metastasis, etc. [52]. *MGAT4C* was identified to be related to animal growth traits [53,54]. *C12orf50* and *C12orf29* are also located in the significant region of 41.78 to 43.05 Mb (~1.27 Mb) in GGA 1. The biological function of *C12orf50* is rarely reported. *C12orf29* played a role in skeletal biology, particularly in the extracellular matrix of cartilaginous tissues [55].

qRT-PCR was performed to evaluate the differences in *KITLG* and *TMTC3* expression levels between stripe pattern and non-stripe pattern RIR chicks. In comparison with chicks of non-stripe pattern, stripe pattern chicks showed significantly higher (*p* < 0.05) expression levels of *TMTC3* in dorsal tissues (Figure 3). *TMTC3* is important for E-cadherin-mediated cell–cell adhesion and plays a role in cell migration, while E-cadherin affects the colonization of melanoblasts/melanocytes; therefore, we speculate that the difference in *TMTC3* expression implies differences in the migration of melanoblasts/melanocytes between chicks of stripe and non-stripe pattern [46]. Compared with darkly pigmented animals of the same breed, light-coated animals possessed lower values in *KITLG* expression level [57,58]. In the present study, the expression level of *KITLG* in striped chicks was higher in value than that in non-striped chicks (*p* = 0.062), which is similar to the previous research results in other species, such as goat, mink, and duck [57,58,59].

## 5. Conclusions

In this study, a genome-wide association study revealed that the genomic region ranging from 41.78 to 43.05 Mb (~1.27 Mb) on GGA 1 is associated with stripe pattern phenotype in pure-bred RIR chicks. Based on genes’ biological functions and differential expression analyses of mRNA, we considered that *KITLG* and *TMTC3* could be candidate genes for the stripe pattern in the RIR chick dorsum. Our results provided a reference to determine molecular mechanisms underlying feather coloration and stripe formation in chicks.

## Figures and Tables

**Table 1:** Primers used in qRT-PCR.

Gene	Primers	Sequence (5′–3′)	Size (bp)	Tm (°C)
*TMTC3*	*TMTC3*-F	TTTGATTGTCTTCAGTCTCCG	132	54
	*TMTC3*-R	CGTTCTGCTACCACAAATCCA		
*KITLG*	*KITLG*-F	AAGAGGCACTTGGCTTCATTAG	138	59
	*KITLG*-R	TTTCTGGTCTGGACTTAGGATG		
*GAPDH*	*GAPDH*-F	ATACACAGAGGACCAGGTTG	130	59
	*GAPDH*-R	AAACTCATTGTCATACCAGG		

**Table 2:** A descriptive summary of significant variants associated with the RIR stripe pattern in GWAS.

Chr.	Position (bp)	N_Sig ^a^	Lead Variant ^b^	*p* ^c^	Genomic Location	Corresponding Genes
1	41785264	1	41785264	7.83 × 10^−6^	exon	*TSPAN19*
1	41799389–41889944	58	41847422	9.24 × 10^−7^	intron; exon; downstream	*ENSGALG00000044478*
1	41892428	1	41892428	3.89 × 10^−6^	Intergenic	*ENSGALG00000044478*-*ALX1*
1	41893987–41921738	18	41916556	1.06 × 10^−7^	upstream; intron; exon; downstream	*ALX1*
1	41902222–41911298	7	41902973	3.89 × 10^−6^	upstream; downstream	*ENSGALG00000047575*
1	41924948–42155127	180	42062678	1.57 × 10^−7^	intergenic	*ALX1*-*RASSF9*
1	42156048–42190437	19	42156048	1.91 × 10^−6^	upstream; exon; intron; downstream	*RASSF9*
1	42198934–42201800	3	42198934; 42200190	3.89 × 10^−6^	intergenic	*RASSF9*-*NTS*
1	42204316–42225096	13	42207440	9.81 × 10^−7^	upstream; intron; downstream	*NTS*
1	42226797–42241774	5	42232409	3.89 × 10^−6^	intergenic	*NTS*-*MGAT4C*
1	42247263–42362279	116	42305962; 42318478	2.25 × 10^−7^	upstream; intron; downstream	*MGAT4C*
1	42363559–42380754	11	42363559; 42372167	1.91 × 10^−6^	intergenic	*MGAT4C*-*ENSGALG00000045907*
1	42387035–42392260	4	42387035	1.91 × 10^−6^	upstream; downstream	*ENSGALG00000045907*
1	42395470–42402424	3	42402424	1.91 × 10^−6^	intergenic	*ENSGALG00000045907*-*ENSGALG00000053372*
1	42417397–42483449	14	42466857	4.83 × 10^−8^	exon; intron; upstream	*ENSGALG00000053372*
1	42484399–42808126	237	42484399	1.56 × 10^−5^	intergenic	*ENSGALG00000053372*-*C12orf50*
1	42808720–42827406	22	42816606	1.91 × 10^−6^	upstream; intron; exon; downstream	*C12orf50*
1	42828049–42836552	13	42835185	4.67 × 10^−7^	upstream; intron; exon	*C12orf29*
1	42837178–42854277	17	42839207	4.73 × 10^−7^	upstream; intron; exon	*ENSGALG00000049176*
1	42857947	1	42857947	2.14 × 10^−5^	intergenic	*ENSGALG00000049176*-*ENSGALG00000051263*
1	42861965–42872432	10	42861965	9.24 × 10^−7^	upstream; intron; downstream	*ENSGALG00000051263*
1	42872979–42883280	14	42877886	9.24 × 10^−7^	exon; intron	*ENSGALG00000011177*
1	42884076–42950258	74	42905449; 42926288	9.24 × 10^−7^	upstream; intron; exon; downstream	*TMTC3*
1	42953794–42977208	12	42973895	1.91 × 10^−6^	intergenic	*TMTC3*-*KITLG*
1	43028225–43047548	2	43047548	3.89 × 10^−6^	intron	*KITLG*
4	21698048	1	21698048	1.60 × 10^−6^	intergenic	*ENSGALG00000048717*-*GASK1B*
25	3002653	1	3002653	7.25 × 10^−6^	upstream	*KCNN3*

^a^ The number of significant variants with *p* < 3.07 × 10^−5^, ^b^ The SNP with the smallest p at the position, ^c^ The *p* of lead variant.

**Table 3:** Known genes associated with a stripe pattern of RIR chicks in GWAS.

Association Genes	Position (bp)	Full Name	Biological Functions
*KITLG*	GGA1 43015486–43066975	*KIT* ligand	Melanoblasts/melanocytes proliferation, differentiation, migration, colonization, melanin production, gametogenesis, and hematopoiesis [32,33,34,35,36,37].
*TMTC3*	GGA1 42888363–42945679	Transmembrane and tetratricopeptide repeat containing 3	Cellular adherence, cell migration, and embryogenesis [45,46].
*TSPAN19*	GGA1 41773256–41785441	Tetraspanin 19	Plasma inhibin B levels [47].
*ALX1*	GGA1 41898277–41919541	*ALX* homeobox 1	Effect craniofacial development and related to beak shape in Darwin’s finches [48].
*RASSF9*	GGA1 42160804–42190042	Ras association domain family member 9	Regulating tumor proliferation and maintainepidermal homeostasis [49,50,51].
*NTS*	GGA1 42207171–42220099	Neurotensin	Regulatory of the central nervous system and digestive system, and promoting tumor metastasis, etc. [52].
*MGAT4C*	GGA1 42251047–42358204	*MGAT4* family member C	Related to animal growth traits [53,54].
*C12orf50*	GGA1 42813465–42822840	*C12orf50* homolog	Unclear
*C12orf29*	GGA1 42829927–42836694	*C12orf29* homolog	Skeletal biology [55].

## Data Availability

The DNA sequencing data for this study can be downloaded from the China National GeneBank (Accession numbers: CNP0003100).

## Supplementary Materials

The following supporting information can be downloaded at: https://www.mdpi.com/article/10.3390/genes13091511/s1, Table S1: A summary of the whole-genome sequencing data; Table S2: The number of variants on each chromosome before and after filtering; Table S3: The significant variants associated with stripe pattern in GWAS.
